# Understanding the mechanical limitations of the performance of soft X-ray monochromators at MAX IV laboratory

**DOI:** 10.1107/S1600577520000843

**Published:** 2020-02-19

**Authors:** Peter Sjöblom, Gabriela Todorescu, Samuli Urpelainen

**Affiliations:** a MAX IV Laboratory, Fotongatan 2, 225 94 Lund, Sweden; b Nano and Molecular Systems Research Unit, PO Box 3000, FI-90014 University of Oulu, Finland

**Keywords:** monochromator, ultra-high resolution, fixed focus constant, dispersion, motion

## Abstract

MAX IV is a fourth-generation, or diffraction-limited, synchrotron light source with a number of state-of-the-art beamlines. The performance of a beamline is, to a high degree, set by the energy resolution it can achieve, which in turn is governed to a large extent by the monochromator. During the design phase of a monochromator, the mechanical requirements must be fully understood and met with margin. During commissioning, the performance must be verified and optimized. In this paper, six soft X-ray monochromators at MAX IV beamlines (Bloch, Veritas, HIPPIE, SPECIES, FinEstBeAMS and SoftiMAX) are examined with a focus on their resolving power, energy range and the time required to change measurement range, as those parameters are dependent on each other. The monochromators have a modern commercial design, planned and developed in close collaboration with the vendors. This paper aims to present the current status of the commissioning at MAX IV with emphasis on elucidating the mechanical limitations on the performance of the monochromators. It contains analysis of the outcome and our approach to achieve fast and high-resolution monochromators.

## Abstract 2

At MAX IV, a fourth-generation, or diffraction-limited, synchrotron light source, six soft X-ray monochromators (Bloch, Veritas, HIPPIE, SPECIES, FinEstBeAMS and SoftiMAX beamlines) are examined with a focus on their resolving power, energy range and the time required to change measurement range. When measuring the mechanical resolution defined by angular vibrations, FinEstBeAMS shows a resolving power *R* of better than 30000 for all studied *c*_ff_ values, while, for HIPPIE, the resolving power for *c*_ff_ = 2.25 and higher and for energies lower than 1 keV is better than 100000.

## 1. Introduction   

The requirements of energy resolution or resolving power, *R* = 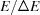, for a synchrotron radiation beamline, and hence the individual components of the entire beamline, are set early in the design process when the optical design is set and optical components chosen. Many of the other design parameters follow as a consequence, including parameters associated with the mechanical motions. For example, resonant inelastic X-ray scattering (RIXS) experiments are especially dependent on high resolution, as losses much smaller than 1 eV must be resolved and hence a resolving power of *R* > 10000 is needed (Willmott, 2011 ▸). As the optics are one of the most expensive parts of the beamline, it is the optics that should be the limiting factor, not the mechanics. The mechanics should provide a sufficient resolution for the measurements, *i.e.* be able to take small enough discrete steps with the optics to change the energy by at least 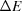 while maintaining the beam centred on the exit slit. On the other hand, other types of experiments, such as X-ray photoemission spectroscopy (XPS) experiments, often require repetitive changing of the photon energy over a wide range to measure different core levels of various elements during, for instance, a chemical reaction (Nonaka *et al.*, 2012 ▸; Müller *et al.*, 2015 ▸; Khalid *et al.*, 2010 ▸). Therefore a sufficiently rapid change of measurement range, *e.g.* the *K* edge of carbon at approximately 284 eV to the *L* edge of copper at approximately 1097 eV, must also be allowed.

In addition, X-ray absorption spectroscopy (XAS), which is a standard technique at most soft X-ray beamlines, requires both a precise scanning of photon energy and sufficiently high scanning speed.

The parameters that limit the speed are also the parameters that limit the resolution, and there is a trade-off between these two requirements. High speed is less of an issue with servo motors compared with stepper motors. For example, the SX700 monochromator, introduced by Petersen & Baumgärtel (1980 ▸), from MAXLAB’s decommissioned beamline I311, was capable of changing the energy from 60 eV to 1000 eV in 12 s. With stepper motors, the same change can take several minutes.

In this paper, the present status of the plane-grating monochromators (PGMs) at MAX IV is reviewed through a combination of measurements and simulations. The review focuses on the PGMs fabricated by Toyama (installed at the Bloch, Veritas, HIPPIE and SoftiMAX beamlines) and by FMB Berlin [installed at the SPECIES (Urpelainen *et al.*, 2017 ▸) and FinEstBeAMS (Pärna *et al.*, 2017 ▸)].

The HIPPIE and Veritas PGMs are identical, except for the optics, while those of SoftiMAX and Bloch have many similarities. The FinEstBeAMS PGM has the same design as SPECIES except for their different choices of angular encoders and angular ranges.

For FinEstBeAMS, a measured stepscan at 400 eV for *c*_ff_ ranging from 1.5 to 20 shows the mechanical behaviour with resolving power *R* = 30000. For Veritas, the measured step­scans shows the mechanical behaviour with resolving power *R* = 80000. When measuring the mechanical resolution defined by angular vibrations for the entire range of energies that the monochromators are intended to operate over, FinEstBeAMS shows a resolving power *R* better than 30000 for all studied *c*_ff_ values and all energies, and for low energies and high *c*_ff_ values the resolving power is improved several times. For HIPPIE the resolving power is better than 50000 over its entire energy and *c*_ff_ range, and for *c*_ff_ = 2.25 and higher the resolution is better than 100000 for energies lower than 1 keV. The performance of Veritas over its entire energy range is somewhat lower than for HIPPIE even though they both share the same mechanical design, but it should be noted that the Veritas monochromator has not been grouted to the floor, which is most likely one of the main contributors to the difference in performance.

## 2. The relationship between energy and angle in a PGM   

**Figure 1:**
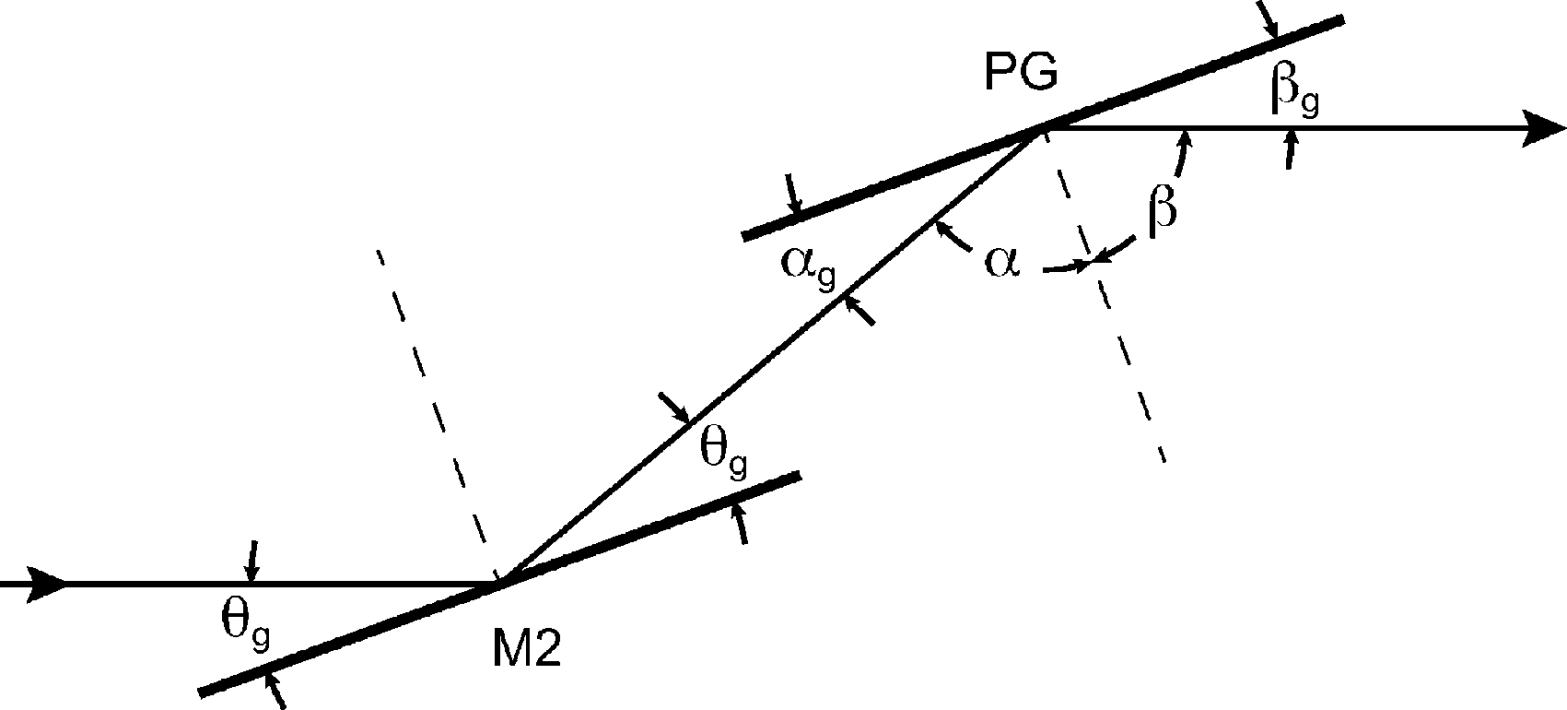
Schematic picture of the geometry of the collimated plane grating monochromator. M2 denotes the mirror and PG the plane grating. The light comes in from the left and exits towards the slit to the right. The incoming and outgoing beams are parallel to each other.

The basic grating equation for diffracted light is
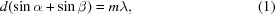
where *d* is the ruling spacing, λ is the wavelength of the light, and *m* is the diffraction order (*m* = 1 throughout this paper). The grating equation describes the beam path through the optics in Fig. 1 ▸ where α and β are the angle of the incoming and outgoing light with respect to the normal of the grating surface, respectively. Note that the 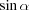 and 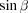 terms are additive since the angle β is measured on the opposite side of the surface normal compared with the angle α.

**Figure 2:**
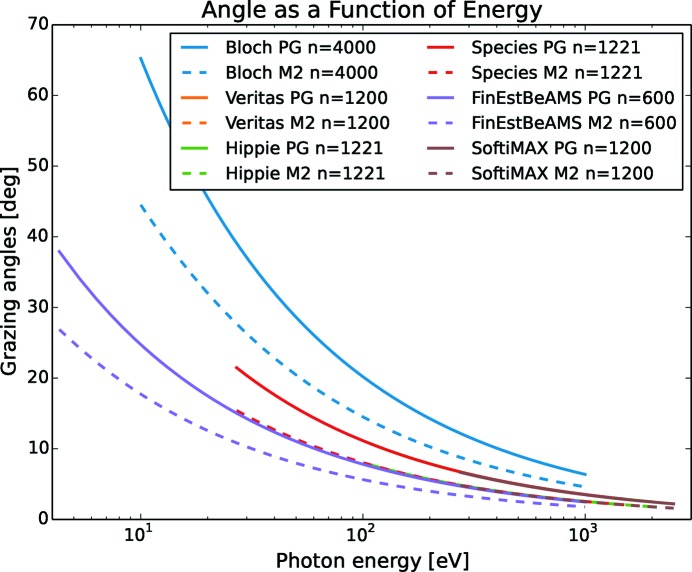
Grazing angles for monochromator optics as a function of photon energy for six different soft X-ray beamlines with their grating. Bloch is coinciding with the FinEstBeAMS line. M2 denotes mirror, PG denotes grating, while *n* is ruling lines per millimetre. SoftiMAX is on top of Species while Veritas is behind.

The wavelength of the light in (1) can be converted to energy using the Planck relation
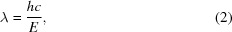
where *E* is the energy of the photons, *h* is the Planck constant and *c* is the speed of light. Introducing the so-called fixed focus condition, *c*_ff_, defined by
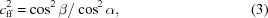
as described by Petersen (1982 ▸) and others, gives a boundary condition for the grating equation. The beauty with imposing the constraint is that the plane grating focal distance becomes constant, *i.e.* remains at all times at the stationary exit slit. Furthermore, in order to keep the light entering and exiting the monochromator parallel to each other (to avoid moving the exit slit), an additional mirror (M2) is added to the beamlines. In order to satisfy all these conditions the entrance (and exit) angle of M2 (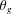, with respect to the mirror surface) needs to be
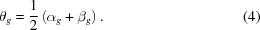
These boundary conditions (Mobilio *et al.*, 2014 ▸) are imposed on the grating equation to set a unique α and β for every λ for a given diffraction order and fixed focus constant (*c*_ff_). If the grating equation (1), fixed focus condition (3) and basic trigonometric relations are used, the wavelength is set by
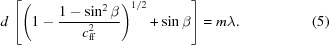
Solving for 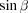 and rearranging the terms gives the quadratic equation
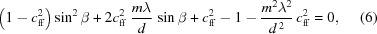
which can be solved for 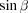. This gives the angle β with respect to the grating surface normal a negative sign. The negative sign indicates that the angle is measured to the opposite side of the normal, when compared with the incoming beam. Using the fixed focus relation, α can be found with
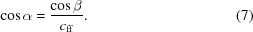
The angle α will have a positive sign and is again measured with respect to the grating surface normal. The grazing angles are then simply
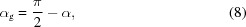


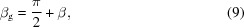
and for M2
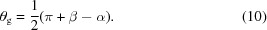
In Fig. 2 ▸, the grazing angles of the grating and mirror are calculated as a function of photon energy using (9) and (10) for six of the soft X-ray beamlines. Each PGM is designed to host two to three gratings. The corresponding motion of the mirror to the grating is indicated by giving the same ruling density *n* (*n* = 1/*d*) with the unit lines per millimetre in the legend, even though the mirror does not have any rules.

**Figure 3:**
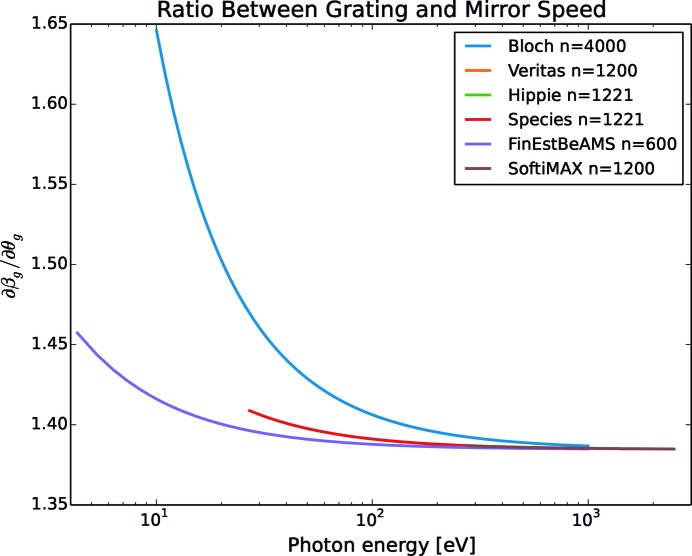
The ratio between grating and mirror speed, 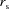, is constant for high energies but changes for lower energies. The plot shows the ratio for *c*_ff_ = 2.25, but will change for other *c*_ff_ values.

From Fig. 2 ▸, it is clear that the gratings have to make a larger angular movement than the mirrors for the same change in energy. The gratings are therefore limiting the time it takes to change the measurement range if the mirror and grating motor speed is set to the same value. As a consequence, the mirror speed can be reduced to allow the mirror and grating to arrive at the same energy at the same time. In this way, the stress of the mirror mechanics is somewhat relaxed. The speed ratio with respect to energy, 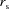, is found by dividing the derivatives of the grazing angles, *i.e.*

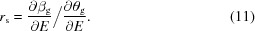
In Fig. 3 ▸ the ratio between grating and mirror speed is plotted. It is close to a constant value for high energies independent of beamline and grating line density but starts to deviate for lower energies. For energies below 500 eV, the speed ratio begins to increase rapidly. For cases where the motor speed cannot be adjusted during movements, a speed ratio of 1.39 for the commonly used *c*_ff_ = 2.25 is a reasonable choice. However, when advanced features, such as continuous scans, are introduced, the nonlinearities of the speed ratio needs to be addressed.

**Figure 4:**
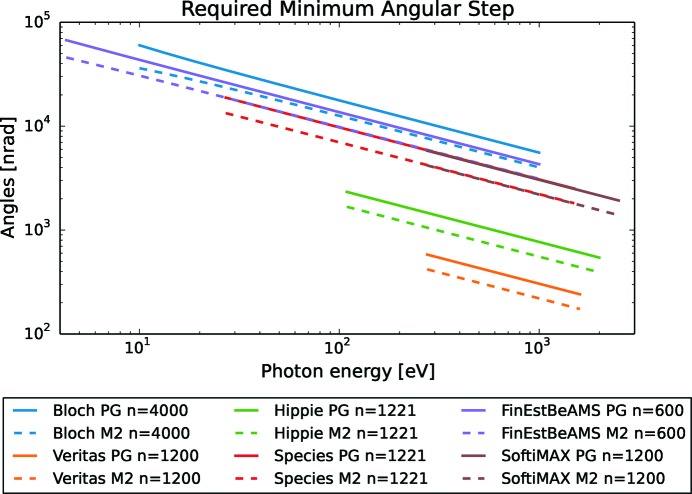
To provide the required energy resolution, 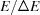, the system must be able to take small enough steps to change the angle. The plot shows the required angular steps for the optics for different energies.

The smallest step in grazing angle needed to yield the required PGM resolving power while maintaining the beam fixed at the exit slit can be calculated with (9) and (10) and is shown in Fig. 4 ▸. For the same small step in energy, the monochromator must reliably be able to move the mirror in smaller angular steps than the grating. Also, the requirements, for both the mirror and grating, increase with higher energies. Both the mechanical suspension of the optics as well as the electronics in the angular encoders should meet this angular requirement with a safety margin, say at least with a factor of five, as the redesign of manufactured systems is costly and a safety margin mitigates the reduction in performance over time and hence the need for maintenance. It also gives some headroom for future upgrades of optics, which could lead to even better resolution. The angular encoders in the described monochromators all have better performance than the mechanics and are therefore not the main limiting factor when it comes to describing the motion. Instead, the encoders can, to a large degree, be used to characterize the rest of the system in terms of thermal drift, backlash and elastic deformation of mechanics or noise contribution from, for example, cooling water, mechanical pumps or people walking past the monochromator. Fig. 4 ▸ shows that the Veritas PGM has the most stringent step requirements due to its resolving power design goal of 100000 — a figure often pursued (Jarrige *et al.*, 2018 ▸; Huang & Chen, 2018 ▸; Song *et al.*, 2006 ▸; Weiss *et al.*, 2001 ▸; Follath, 2001 ▸) and denoted ultra-high resolution. For high energies, the mechanics should be able to provide steps as small as 20 nrad to make sure that it is the optics that limits the resolution.

Another conclusion from Figs. 2 ▸ and 4 ▸ is that the requirements on motion vary significantly between the PGMs and therefore need to be addressed individually based on each PGMs individual design goal. Depending on the specific experiments to be performed, different settings for otherwise similar systems should be considered as well as different settings based on different experiments on the same system.

## 3. The influence of the fixed focus constant on energy and angle in PGM stepscans   

It has been shown (Petersen *et al.*, 1995 ▸) that *c*_ff_ = 2.25 gives the optimum grating efficiency for a wide energy spectrum. However, there are situations where it could be an advantage to change the *c*_ff_ and thereby change the distance to the virtual source 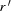 where the light appears to be coming from. The virtual source distance is proportional to the source distance, *r*, as
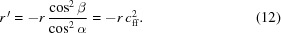
Changing *c*_ff_ can (Follath & Senf, 1997 ▸): (i) give higher energy resolution, (ii) suppress higher orders, (iii) provide a high flux mode, (iv) focus the zeroth order (*c*_ff_ = 1) or (v) change from inside to outside diffraction order (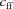 > 1 to 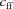 < 1).

These considerations make a variable *c*_ff_ a tempting option. In Figs. 2 ▸ and 4 ▸, the assumption is to use *c*_ff_ = 2.25, but with other values the grazing angles and smallest necessary angular step size will change too.

**Figure 5:**
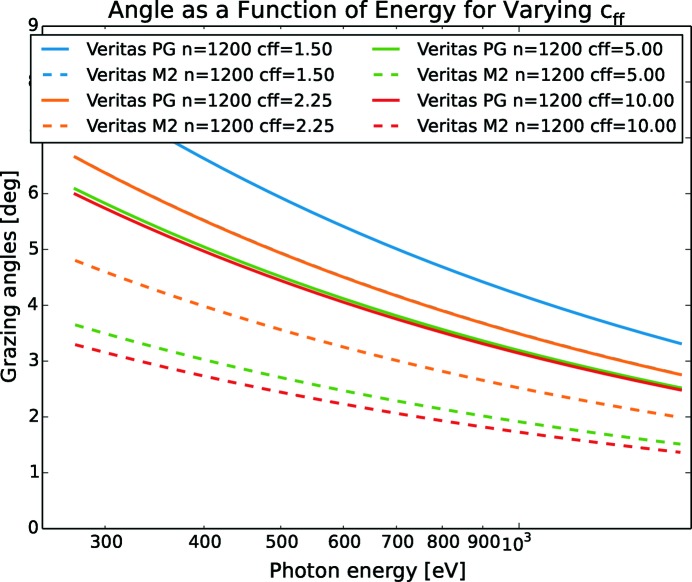
Grazing angles for the Veritas monochromator mirror and grating as a function of photon energy with changing *c*_ff_ value. For high values of *c*_ff_ the angle for incoming light is steeper.

Using the Veritas PGM mirror as an example, Fig. 5 ▸ shows that for a given energy a decreasing *c*_ff_ results in larger grazing angles and, as a consequence, the higher-order content decreases. With higher *c*_ff_ values, the grazing angles become smaller and from (12) it follows that the virtual source appears as more distant and therefore the size of the virtual source also decreases. This leads to a stronger demagnification of the source on the exit slit but at a cost of lower flux as the grating acceptance and grating efficiency decrease as well.

**Figure 6:**
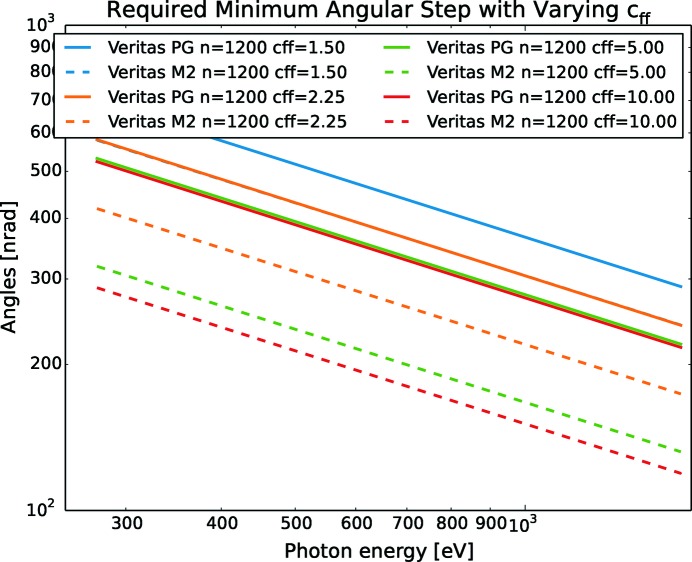
Required angular steps for the Veritas mirror and grating as a function of energy for different *c*_ff_. For high values of *c*_ff_ the required steps become smaller. Also, the mirror needs to take smaller steps than the grating.

In Fig. 6 ▸, the motion of the Veritas mirror required to reach a resolution of 100000 is shown. As the mirror reaches the requested resolution at larger steps for low *c*_ff_ values, higher resolutions would be expected for *c*_ff_ < 2.25 than for 2.25 as the noise is the same regardless of the *c*_ff_ value.

**Figure 7:**
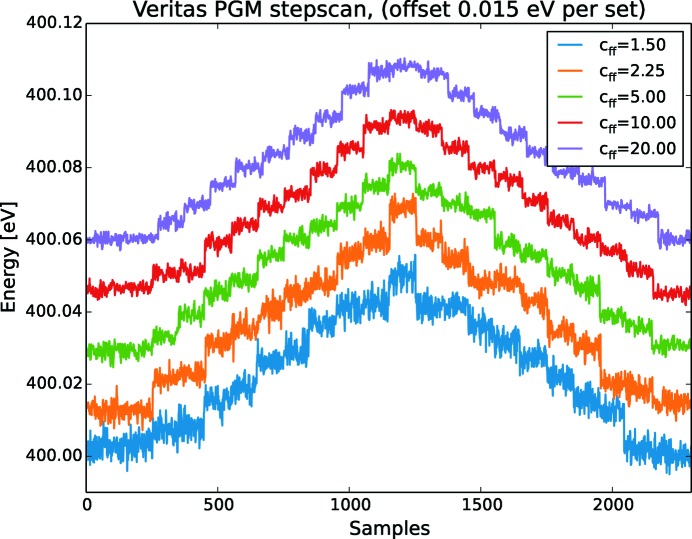
The sampled angular noise in encoder position converted to energy for small energy steps for different *c*_ff_ values at Veritas. Each step is 5 meV and 10 s at 400 eV giving a resolution of 80000. The system behaviour is better at higher *c*_ff_ values. The cooling water is turned off.

**Figure 8:**
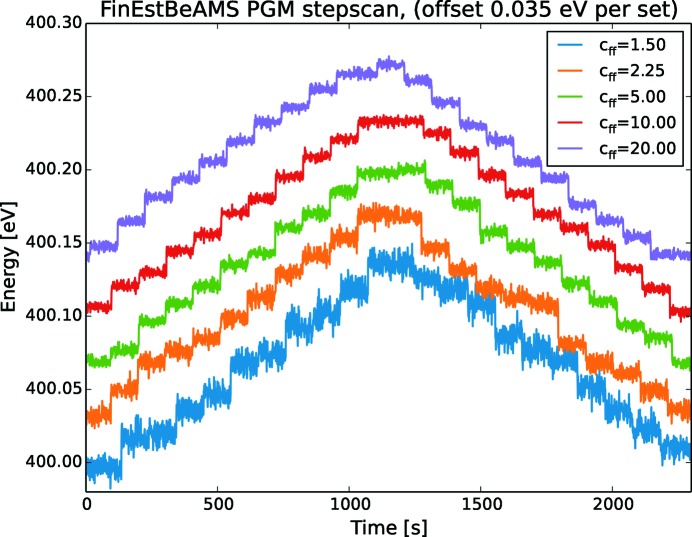
The sampled angular noise in encoder position converted to energy for small energy steps for different *c*_ff_ values at FinEstBeAMS. Each step is 13 meV and 10 s at 400 eV giving a resolution of 30000. The system behaviour is better at higher *c*_ff_ values. There is no concrete under the stone supporting the PGM. The cooling water is turned on.

In Fig. 7 ▸, measurements on Veritas, where the monochromator is performing a stepscan around 400 eV for different *c*_ff_ values, show that the noise content in the anglular encoders, when the angles are converted to energy, have a more stable behaviour for high values of *c*_ff_. Note that all measurements start at 400 eV but are offset by 0.035 eV for clarity. When the *c*_ff_ increases, the steps becomes more and more separated while the noise is suppressed. The same behaviour is found in the FinEstBeAMS monochromator as shown in Fig. 8 ▸. As with Veritas, all measurements started at 400 eV and are offset 0.015 eV for clarity. The Veritas measurement is made with cooling water turned off while for FinEstBeAMS it is turned on.

The increased performance with increased *c*_ff_ is not due to the larger angular steps that need to be taken as the larger steps are found for lower *c*_ff_. The dispersion, 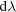, is, for example, found by taking the derivative of (1) or (5), with α as a constant (Peatman, 1997 ▸; Howells, 2009 ▸), remembering that *c*_ff_ is a function of β, which results in
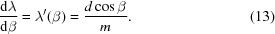
Using the concept of optical path function, *F*, the dispersion is determined by (Peatman, 1997 ▸; Howells, 2009 ▸)
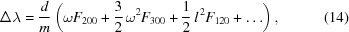
where ω is a position in the dispersive plane and *l* is a position in the sagittal plane. To achieve a high-resolution large dispersion, the most important term to minimize is thus the meridional focus term, 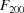.

In the case of a plane grating monochromator, 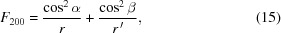
which is minimized for small grazing angels and high *c*_ff_.

It should also be remembered that (13) gives the dispersion per 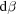 while the actual angle change 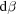 per motor step depends on the suspension of the grating.

Another conclusion from Figs. 7 ▸ and 8 ▸ is that the ability to reproduce a measurement or return to the same energy a second time is very high. If the offset is not added the step­scans are seen to overlap to a high degree. The stepscans are parallel and the scan returns to its initial value regardless of whether it is after a positive or negative step.

Determining the resolution by a stepscan as in Figs. 7 ▸ and  8 ▸ is an arbitrary approach, as there is no stringent way of stating what is a good enough step. Nevertheless, it is fast and intuitive to evaluate noise, tilting, drifting, overshoot and other parameters important in motion, and will therefore serve a purpose here.

**Figure 9:**
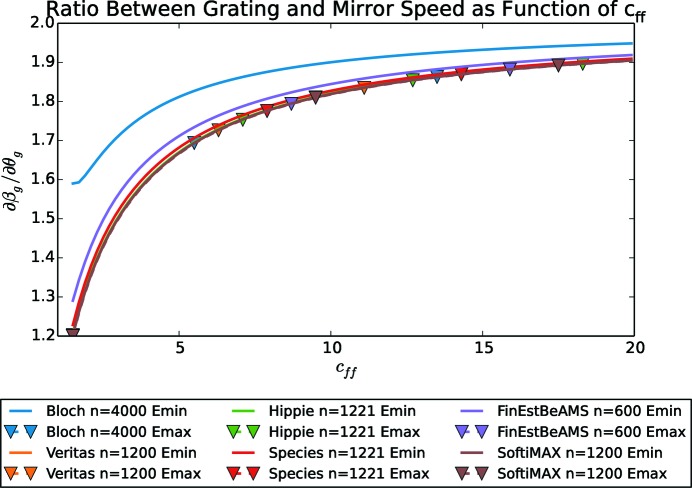
The ratio between grating and mirror speed as a function of *c*_ff_ value. For high values of *c*_ff_ the ratio approaches the value 2. Emax and Emin are the maximum and the minimum energy that beamline can have. For the commonly used *c*_ff_ = 2.25, a speed ratio of 1.39 is often a well balanced choice.

It is also important to note that the speed ratio, 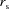, equation (11), is dependent on the *c*_ff_ value, since the angles of the grating and mirror for a specific energy change with *c*_ff_. The speed ratio is shown in Fig. 9 ▸ as a function of *c*_ff_. For each monochromator and its gratings, the speed ratio is shown for two energies, the lowest and the highest energy that beamline with that grating can give. The speed ratio is rather independent of the system, the grating in use and the energy but increases as the *c*_ff_ value increases; for extreme values the speed ratio approaches 2 asymptotically.

## 4. Resolving power as a function of energy and fixed focus constant   

To overcome the arbitrariness in judging what step size is large enough in a stepscan to separate two energies with the aim to determine mechanical resolution, it is better to study the Gaussian full with at half-maximum (FWHM) of the noise in the energy while the monochromator is standing still rather than to measure on a sample. With this reasoning, the mechanical resolution of the monochromator is defined as the average energy divided by FWHM of its noise for that energy, *i.e.* 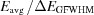, under the assumption that both encoder and motor steps allows finer steps than the amplitude of the noise. For all the tested monochromators, both motor step size and angle step size are much smaller than the noise.

**Figure 10:**
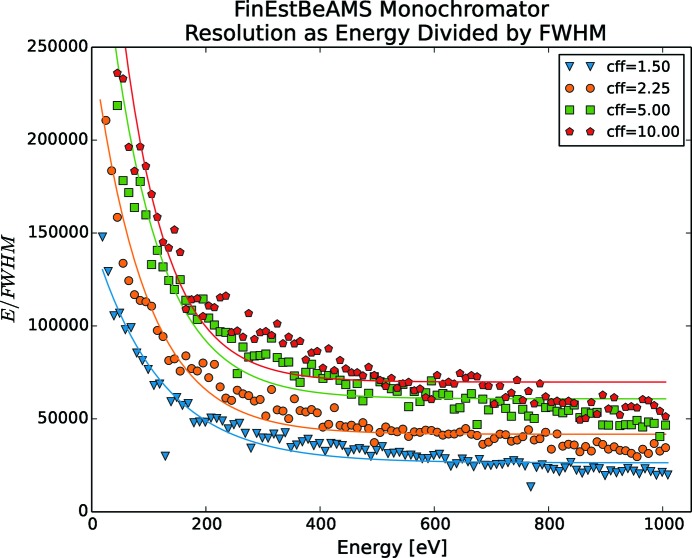
FinEstBeAMS mechanical resolution as function of energy and *c*_ff_. Each energy scan is divided into 100 steps, where the mechanics were standing still. For each measurement series, a power fit is also shown. For best mechanical resolution, a high *c*_ff_ should be used.

**Figure 11:**
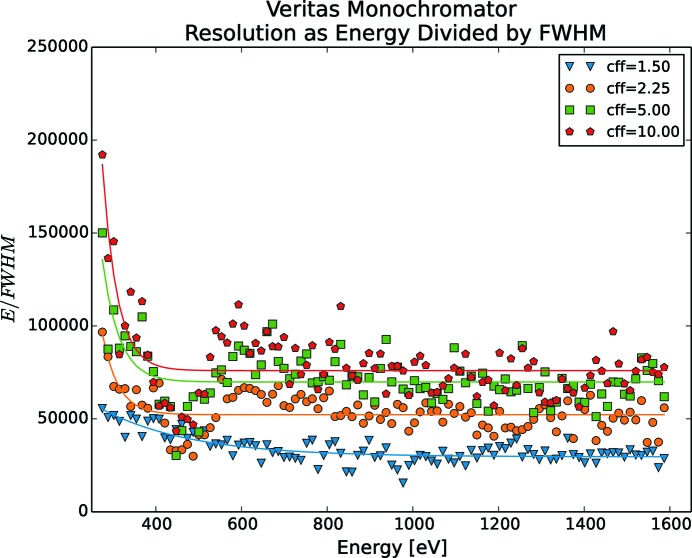
Veritas mechanical resolution as function of energy and *c*_ff_. Each energy scan is divided into 100 steps, where the mechanics were standing still. For each measurement series, a power fit is also shown. For best mechanical resolution, a high *c*_ff_ should be used.

**Figure 12:**
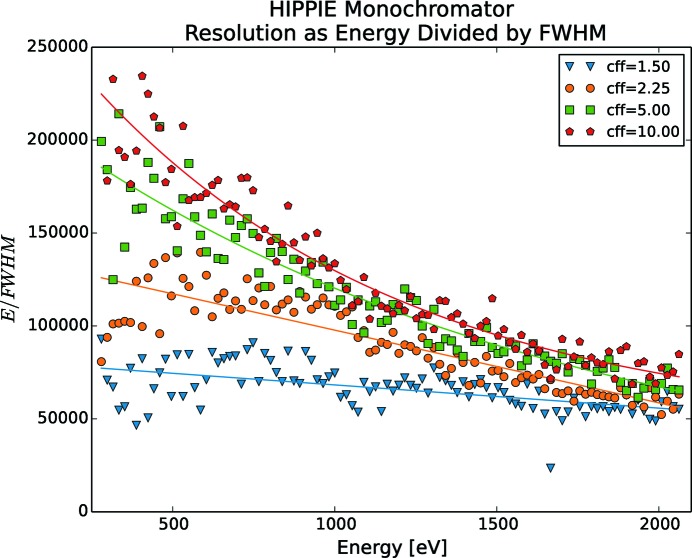
HIPPIE mechanical resolution as function of energy and *c*_ff_. Each energy scan is divided into 100 steps, where the mechanics were standing still. For each measurement series, a power fit is also shown. For best mechanical resolution, a high *c*_ff_ should be used.

**Figure 13:**
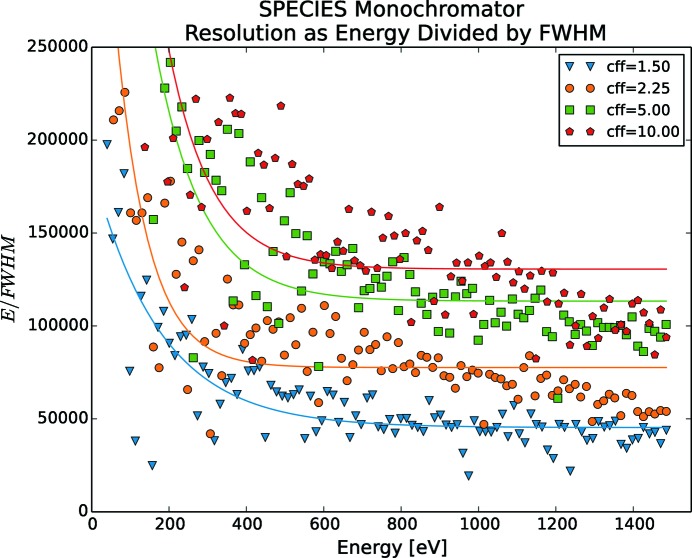
SPECIES mechanical resolution as a function of energy and *c*_ff_. Each energy scan is divided into 100 steps, where the mechanics were standing still. For each measurement series, a power fit is also shown. For best mechanical resolution, a high *c*_ff_ should be used.

Such resolution measurements have been made for FinEstBeAMS (Fig. 10 ▸), Veritas (Fig. 11 ▸), HIPPIE (Fig. 12 ▸) and SPECIES (Fig. 13 ▸), by running a stepscan from their lowest to their highest energies. The energy spectrum was divided into 100 steps and for each step the energy was sampled for 10 s with a 33 Hz rate for five different *c*_ff_ values, namely 1.5, 2.25, 5, 10 and 20. The results for *c*_ff_ = 20 are left out in the figures as the change in performance increased only marginally compared with *c*_ff_ = 10. Measurements are with fixed focus, *i.e.* both mirror and grating are moved for each energy change. The resolution increases for lower energies and rapidly increases for energies below 200 eV. Also, selecting a higher *c*_ff_ results in a higher resolution. As FinEstBeAMS reaches low energies, very high resolution can be achieved. For Veritas, a phenomenon around 450 eV makes the resolution drop, especially for higher *c*_ff_ values. At the time of writing, the root cause of this is unknown, but is suspected to be a lack of concrete under the monochromator plinth, as the otherwise identical monochromator at HIPPIE does not show this behaviour. SPECIES is by far reaching a better mechanical performance than what is required by optics.

**Figure 14:**
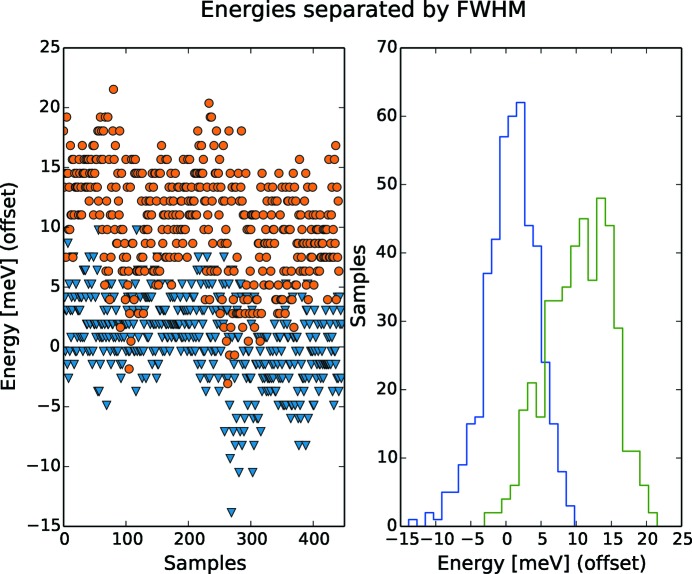
The separation of energy with 2σ is illustrated by offsetting a measured energy to zero and the next energy 2σ above. To the left, energy per sample. To the right, the same data but as a histogram. Data from HIPPIE, *c*_ff_ = 2.25, 694 eV.

To visualize a single step, the left-hand panel of Fig. 14 ▸ shows two energies from the HIPPIE measurement presented in Fig. 12 ▸, where the first energy is centred around 0 eV and the second is centred one FWHM above. The right-hand panel describes the the same data as histograms. A clear separation between the energies is visible.

## 5. PGM optics mechanical angle equations   

**Figure 15:**
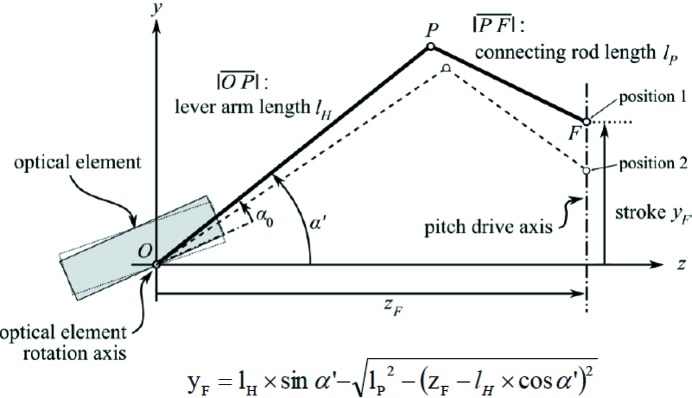
The geometry of SPECIES and FinEstBeAMS monochromators together with the motion law describing the motion of the optics. (Courtesy of FMB Berlin.)

**Figure 16:**
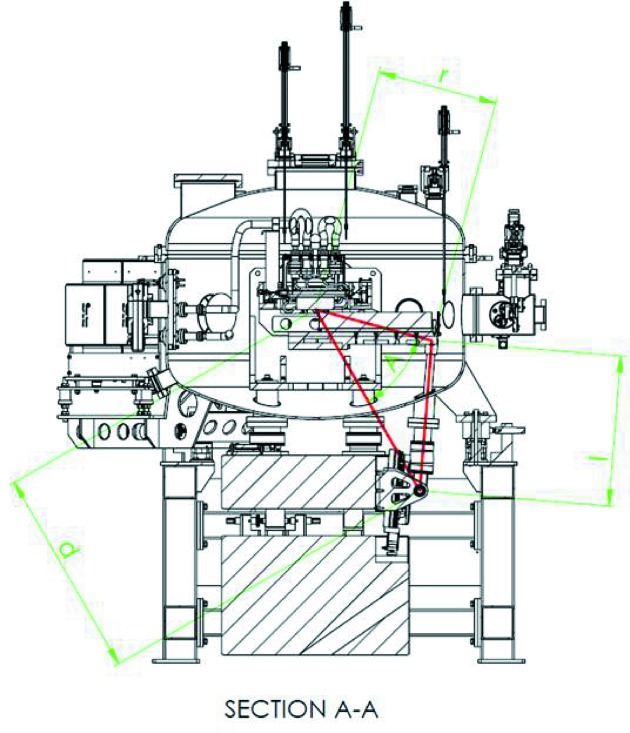
The geometry of HIPPIE, Veritas, Bloch and SoftiMAX monochromators showing that the law of cosine describes the motion of the optics. (Courtesy of Toyama.)

The requirements on the angular resolution give constraints on the mechanics and the motors. The equation describing the mechanical movement of the monochromator for the FinEstBeAMS and SPECIES beamlines is
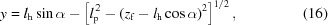
where 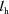 is the lever arm length, 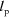 is the connecting rod length, 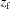 is the driver distance to the axis of rotation, and *y* is the stroke as illustrated in Fig. 15 ▸. For Bloch, Veritas, HIPPIE and SoftiMAX, the equivalent equation is
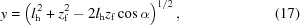
which is the cosine law and shown in Fig. 16 ▸. The parameter’s names and indexes in (16) and (17) are chosen to be the same for all the monochromators.

**Figure 17:**
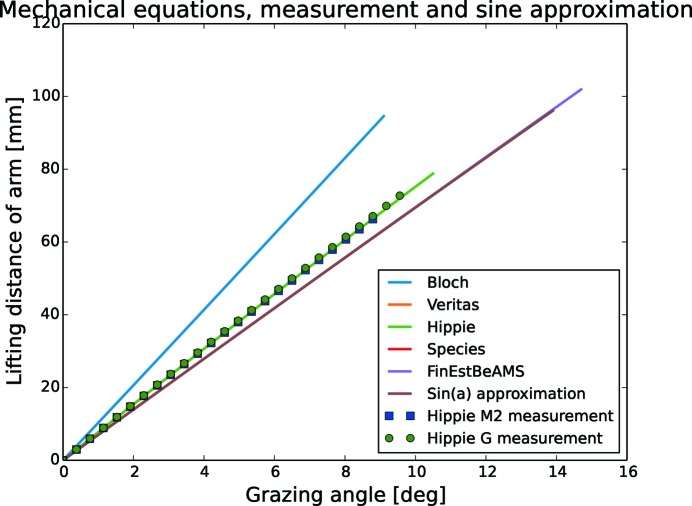
The equations (16) and (17) that describe the grating and the mirror motion can in many cases be replaced by the sine equation for fast and simple engineering purposes.

When equations (16) and (17) are visualized, as in Fig. 17 ▸, it is clear that, for engineering purposes at small angles, the equations for SPECIES and FinEstBeAMS can be well approximated by sinusoids. For Veritas and HIPPIE this is also a good first approximation, while for Bloch the fitting is less accurate meaning that the motion is underestimated with a sine approximation.

Starting with the sine arm approximation and with an expected operation angle of α, the lifting and the lowering of the arm is 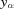 = 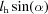. With a threading 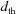 of the screw and a gearbox ratio of *g* turns per turn, the motor needs to rotate 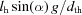 turns to accomplish the motion. With a maximum speed of 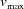 turns per second, the minimum time 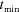 to make a full stroke of the system would be
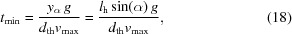
showing the impact of the gearbox and the threading of the screw.

Considering the resolution, the angle each motor step makes the shaft rotate after the gearbox is 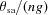 where 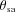 is the full step angle of the motor (usually expressed in degrees), *n* is the level of microstepping and *g* is the gear ratio in the gearbox. Every step causes a lift or a drop of the mechanics by an amount 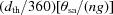, which in turns causes the smallest shift in angle of the optics and therefore determines the resolution of the system according to the mechanical equation as
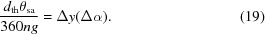
In the case of a sine approximation,
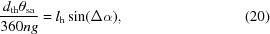
the mechanical requirements to meet the requested resolution can be found as
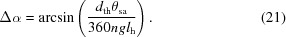
From (19) it is also clear that 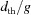 can be described as a function of the smallest change in *y* caused by the smallest change in angle, 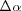, which results in the equation
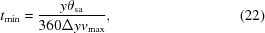
and for a sine approximation
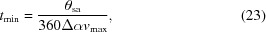
where the minimum time to change from one range to another is a function of the smallest step the system needs to take, 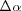.

**Figure 18:**
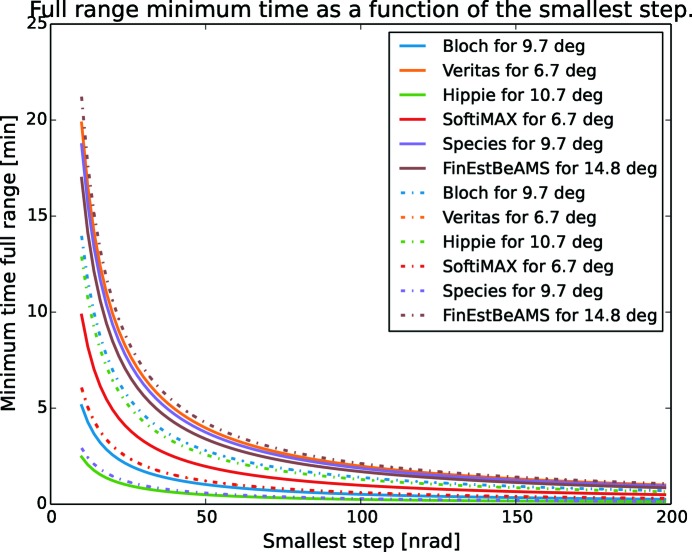
The time it takes to run from zero to maximum angle as a function of the smallest step the system can take. Dotted lines indicate a sine approximation. Note that SoftiMAX and FinEstBeAMS are almost overlapping each other.

In Fig. 18 ▸, the minimum time as a function of the smallest step is shown by the solid lines together with the dotted lines representing a sine arm approximation. When small steps are needed, the time for a full stroke becomes large and must be regarded in the early design. The time to resettle from a measurement in the low-energy region to a measurement in the high-energy region takes several minutes. During this time no experiments can be performed. This is a considerable drawback in cases where the sample under investigation is sensitive to beam damage, the experiment itself is time sensitive or the measurement range needs to be changed often, which would mean that the time the system is available for sampling is reduced.

## 6. Influence of encoder, motor, gearbox and driver   

The encoders used for the positioning of the mirrors and gratings in the monochromators at MAX IV are all angular optical encoders, but differ slightly in their readout. Bloch, HIPPIE, Veritas and Softimax use incremental encoders with 24 nrad resolution while FinEstBeaMS uses absolute encoders with 1.5 nrad resolution and SPECIES uses an analog encoder with 11 µA signal which is digitized to 109 nrad. All monochromators except the one at SPECIES also have absolute linear encoders on the lifting mechanism with 1 nm resolution, but those encoders are not used for positioning. They will, in a later state of commissioning, be connected to the PLC system to be a part of protection against the possibility of a collision between grating and mirror mechanics under certain conditions.

**Figure 19:**
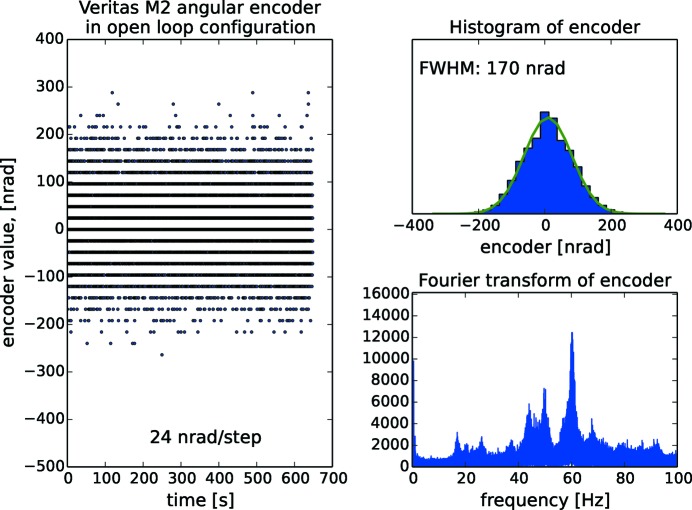
Open loop control of the Veritas monochromator mirror with histogram and Fourier analysis of incremental angular encoder signals. The signal is sampled for 10 min with 4.5 ms interval. The Gaussian FWHM is 170 nrad and in the spectrum vibrations peaks at 20 Hz originate from the floor while the peaks above 50 Hz originate from pumps and ventilation.

**Figure 20:**
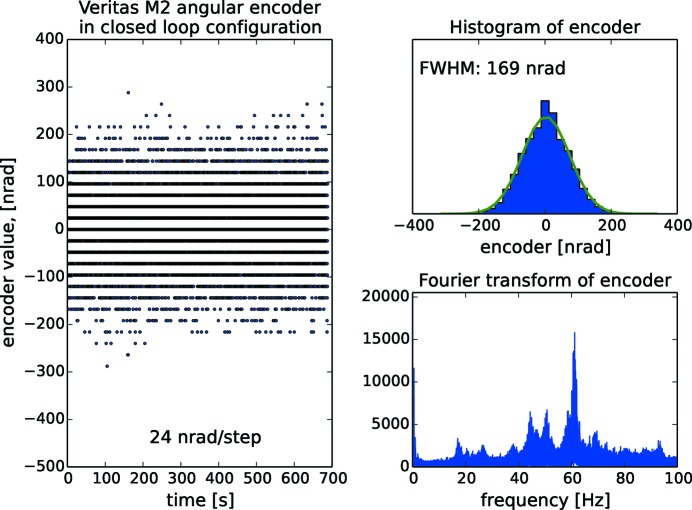
Closed loop control of the Veritas monochromator mirror with histogram and Fourier analysis of incremental angular encoder signals. The signal is sampled for 10 min with 4.5 ms interval. The Gaussian FWHM is 170 nrad and in the spectrum vibrations peaks at 20 Hz originate from the floor while the peaks above 40 Hz originate from pumps and ventilation.

**Figure 21:**
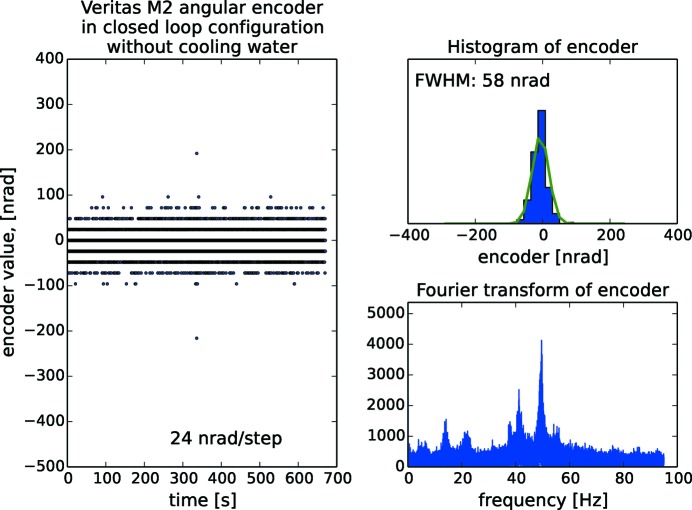
Closed loop control of the Veritas monochromator mirror with histogram and Fourier analysis of incremental angular encoder signals when the cooling water and guard vacuum pump is turned off. The signal is sampled for 10 min with 4.5 ms interval. The Gaussian FWHM is 58 nrad and in the spectrum vibrations peaks at 20 Hz originate from the floor while the peaks above 40 Hz originate from pumps and ventilation.

As all the angular encoders operate with nrad resolution, the encoder signal is a means to characterize the entire monochromator rather than only the encoder itself. On Veritas, which we have picked as an example for the other beamlines, the analysis is done in open loop operation (Fig. 19 ▸), in closed loop operation (Fig. 20 ▸) and in closed loop operation with cooling water and guard vacuum pump turned off (Fig. 21 ▸). The Gaussian FWHM for Veritas is 170 nrad in both open and closed loop operation with cooling water flowing. Also, the fast Fourier transform (FFT) reveals that the same frequency content is present in both situations, *i.e.* closed loop operation does not necessarily add vibrations. The frequency content reveals peaks at 20 Hz arising predominantly from floor vibrations. Contributions above 40 Hz originate from other sources: there is a temporary mechanical guard vacuum pump separating the cooling water tube from the rest of the vacuum chamber which will be later replaced by a turbo pump; there are also fans for cooling the turbo pumps directly mounted on the monochromator vacuum chamber; the air ventilation system, located inside the optical hutch, regulates the temperature to 24 ± 0.1°C, the same temperature as in the ring hall. These sources of vibrations make the monochromator resonate at its eigenfrequencis. Turning off the cooling water reduces the FWHM to 58 nrad, or to about one-third compared with when cooling water is flowing. HIPPIE is showing the same behaviour as Veritas with two frequencies around 20 Hz and a number of resonances above 50 Hz as well as a FWHM at 66 nrad with cooling water turned off. In the case of FinEstBeAMS, the lowest frequency is found at 34 Hz, *i.e.* higher that the rest of the systems. The behaviour of the gratings is better than the mirrors. Resonance frequencies start at 55 Hz for Veritas and HIPPIE and 100 Hz for FinEstBeAMS. The overall conclusion is that the major contributor to noise is the turbulent flow of cooling water in the optics.

The time it takes to make a large motion is very much dependent on the selection of the motor and the electronics driving it. For all the new monochromators at MAX IV, two phase hybrid stepper motors are selected. The reason for using stepper motors over, for example, servo motors is the ability of stepper motors to stand still over long periods of time with low vibration, a property required for long XPS or RIXS experiments, for example. A big disadvantage is their low speed, another that the motors have discrete steps, which on the other hand is easily mitigated by microstepping. One thing to note is that, for a given resolution determined by gear ratio, threading and microstepping, a high level of microstepping will reduce the time to change range only if 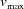 is dependent on the level of microstepping.

At MAX IV, the chosen motor driver is IcePAP (Janvier *et al.*, 2013 ▸), which is a voltage-type driver. Instead of a step response that sends the rotor uncontrolled to a new position, the current step is sinusoidal and hence allows the rotor to follow the magnetic field to its new position and settle there. In this way, the maximum speed is insensitive to the level of microstepping and vibrations are avoided. Microstepping is thus a better choice than the use of a gearbox to increase resolution as it does not affect motion speed.

At MAX IV the soft X-ray monochromators are all using similar motors from Oriental. The time constant τ = *L*/*R* for PK245MD15B is τ = 5.1/2.1 = 2.4 ms; for PK268-03B, τ = 6.4/2.0 = 3.2 ms; for PKP264MD28B-L, τ = 6.0/1.2 = 4.9 ms; and for PK264DB, τ = 0.6/0.3 = 2.0 ms, where *L* is in mH and *R* is in Ω as stated in their data sheets. The ratio of load inertia to motor inertia is selected to be equal or slightly larger than one to ensure a smooth behaviour over short and quick motions.

One way of achieving faster stepper motion is to use viscous dampers (Lanchester dampers) mounted on the motor shaft. The inertia of the viscous material tends to make it rotate at a constant speed together with the motor. It damps, through friction, any changes in speed, such as oscillation and vibrations. Anecdotally, at Bloch, during commissioning, the dampers on the PGM needed to be removed temporarily, to allow access to other mechanical details. When the motors for the pitch angles were run, the speed had to be reduced to a fifth without dampers compared with that with dampers. The optimal ratio (Kenjo & Sugawara, 1994 ▸; Acarnley, 2002 ▸) between the motor and damper housing inertia to the viscous inertia of the damper is shown to be 4.

The selection of gearbox, threading, arm lengths, type of joints and so forth is a tradeoff between design parameters such as backlash, torque requirements and manufacturing tolerances and the influence of them on a system is, for example, highlighted by Zhang *et al.* (2018 ▸). Some kind of energy adjustment or calibration to map the actual energy to theory and calculated angles is unavoidable.

## 7. Influence of cooling water on energy and angle in a PGM   

**Figure 22:**
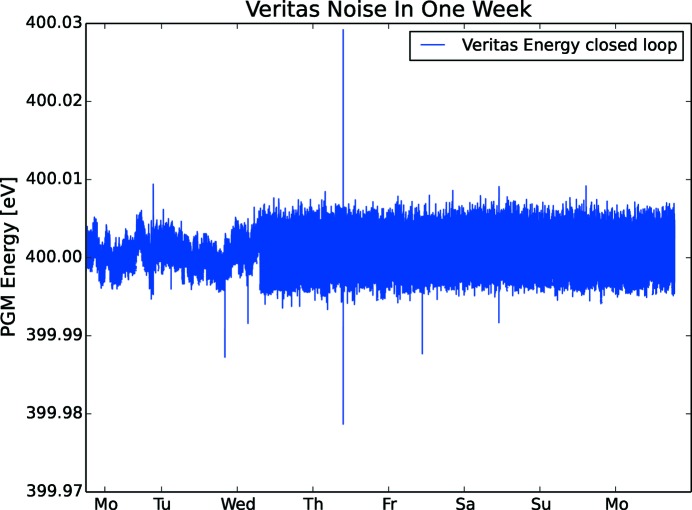
Veritas PGM in closed loop was standing still for one week while the energy was sampled. Mid-week, the cooling water was turned on increasing the noise from 3 meV to 7 meV.

With cooling water flowing in the optics, the resolution drops due to vibrations from water turbulence, making the resolution a function of water pressure. On the other hand, without stable temperature on flowing cooling water, the beam will move [see Peatman (1997 ▸), compare 0.5°C with 0.05°C]. To illustrate the influence of cooling water on noise, a long duration energy measurement of the stationary optics was made on Veritas (Fig. 22 ▸). The measurement ran for seven days. On the third day, the cooling water in the optics was turned on and clearly contributed to the overall noise level, which increased from about 3 meV to 7 meV and hence reduced the possible resolution of the PGM. The water pressure was 4.4 bar and the flow 7.8 l min^−1^ for M2 and 4.2 l min^−1^ for the grating. The water temperature was 26.5°C. (For HIPPIE, the pressure was 5.0 bar, M2 flow 7.5 l min^−1^, PG flow 3.4 l min^−1^ and temperature 26.5°C. For FinEstBeAMS, the pressure was 3.9 bar, M2 flow 5.7 l min^−1^ and temperature 26.5°C.) The cooling water temperature variation is 0.1°C as measured on the outlet of the optics. The water flow is deliberately made turbulent to ensure efficient cooling of the optics, which otherwise would quickly be heated and create bumps in the surface and hence add aberration and drift of the light spot. One important conclusion is that water pressure should be kept at the required minimum to provide cooling for the optics, if high resolution is needed. Also, there is no concrete under the stone supporting the PGM, which will reduce noise when in place.

Turbulent water has been identified (Strocov *et al.*, 2010 ▸) to be a significant source of vibrations and has previously been dealt with by adjusting the water flow depending on the heat load, *i.e.* the *K*-value of the insertion device. Another approach is to use gravitational flow (Khalid *et al.*, 2010 ▸).

## 8. Overview of systems   

To provide an overview of MAX IV’s soft X-ray systems, key parameters are summarized in Table 1 ▸ showing the present status. They are used to present the plots in the paper but should not be regarded as static as all beamlines at MAX IV are going through commissioning and are hence constantly improved and changed to meet new requirements.

## 9. Conclusions   

To achieve a high-resolution beamline, each element in the beamline must fulfil their specification. In this paper, key parameters of six soft X-ray plane grating monochromators at MAX IV Laboratory have been simulated and measured to determine if they meet the mechanical requirements of resolving power and motion speed. Noise from encoders has been used to determine the resolving power as a function of energy and *c*_ff_, and turbulent cooling water has been identified as a major contributor to noise. Parameters such as speed, resolution and time to change measurement range have been characterized through equations and measurements.

## Figures and Tables

**Table 1:** Overview of beamline parameters

	Bloch	Veritas	HIPPIE	SPECIES	FinEstBeAMS	SoftiMAX
Resolving power^*a*^	10000	100000	40000	10000	5000	10000
Energy range (eV)	10–1000	275–1600	109–2000	27–1500	4.3–1000	275–2500
Grating lines (mm^−1^)	92	1200	1221	250	92	300
	800	2400	2400	1221	600	1200
	2400					
	4000					
Full step (°)	0.9	0.9	0.9	1.8	0.9	0.9
Microstepping	2	8	2	16	2	2
Motor speed^*b*^ (rev/s)	25	10	30	15	25	20
Motor^*c*^	245	245	245	268	264MD	264DB
Damper^*d*^	D4	D4	D4	–	D6	D6
Gearbox ratio	100	100	100	100	100	50
Angular encoder (nrad)	24^*e*^	24^*e*^	24^*e*^	109^*f*^	1.5^*g*^	24^*e*^
Grating stroke (°)	9.2	6.6	10.6	9.5	13.3	6.7
Mirror stroke (°)	6.6	4.8	7.7	6.9	9.6	4.8
Time^*h*^ 60–1000 eV (min)	3.1	6.9^*i*^	2.3^*i*^	4.2	3.5	1.7

^*a*^The goal set during design. Actual resolving power has still to be measured. ^*b*^The speed is shown for the grating as it is the speed limiting element. ^*c*^Same motor for both M2 and grating pitch. Motor manufacturer is Oriental and the models are PK245MD15B, PK268-03B, PKP264MD28B-L and PK264DB. ^*d*^Damper models are: D4CL-5.0F and D6CL-8.0F for FinEstBeAMS and D6CL-6-3F for SoftiMAX. ^e^Renishaw two head incremental averaged with 24 nrad per step for each head. ^*f*^Four head RON905UHV analog 11 µA_pp_ sin/cos signal from 36000 lines fed to EXE660B 1600 times interpolator and digitizer results in 109 nrad resolution. At this time only one head is used. Encoder signal error compensated with Heydemann Correction. ^*g*^Renishaw 32-bit REXA absolute encoder with 1.5 nrad resolution. One out of two heads are used. ^*h*^Measured time to change energy from 60 to 1000 eV. (SoftiMAX is an estimation.) ^*i*^The time if 60 to 1000 eV would have been inside the intended range of operation.
